# Editorial: Immune responses at barrier tissues: insights from synthetic biology in therapeutics, diagnostics, mechanisms, and beyond

**DOI:** 10.3389/fimmu.2025.1621688

**Published:** 2025-06-02

**Authors:** Siheng Chao, Naoki Iwanaga, Hernan Felipe Peñaloza, Nicola Ivan Lorè, Kong Chen

**Affiliations:** ^1^ Division of Pulmonary, Allergy, Critical Care, and Sleep Medicine, Department of Medicine, University of Pittsburgh, Pittsburgh, PA, United States; ^2^ Department of Respiratory Medicine, Nagasaki University Hospital, Nagasaki, Japan; ^3^ Millennium Institute on Immunology and Immunotherapy, Santiago, Chile; ^4^ Facultad de Ciencias Biológicas, Pontificia Universidad Católica de Chile, Santiago, Chile; ^5^ Departamento de Laboratorios Clínicos, Escuela de Medicina Facultad de Medicina, Pontificia Universidad Católica de Chile, Santiago, Chile; ^6^ Emerging Bacterial Pathogens Unit, Division of Immunology, Transplantation and Infectious, IRCCS Ospedale San Raffaele, Milan, Italy

**Keywords:** immune response, barrier tissue, therapeutics, diagnostics, mechanisms

Barrier tissues separate the external environment from the internal environment of the human body. These specialized structures serve not only as passive physical barriers but also as dynamic regulators of molecular and cellular traffic, thereby playing a critical role in maintaining physiological homeostasis. Barrier integrity is essential across multiple systems, including the respiratory, urinary, gastrointestinal, and sensory systems. In pathological states, disruption of barrier function permits the translocation of inappropriate or excessive molecular species, which can precipitate aberrant immune activation, often skewed toward pro-inflammatory responses. Such immune dysregulation may exacerbate existing pathology or contribute to the onset of comorbid conditions.

This Research Topic sheds light on the pathophysiology of barrier tissues is disease contexts across multiple organ systems. It emphasizes the underlying mechanisms of barrier tissue dysfunction and the immune system’s role in these processes, while highlighting potential biological models and outlining future clinical research directions to manage diseases with barrier tissues in mind.

The mini review by Simmalee et al. describes the pathogenic roles and effects of inflammatory eosinophils (iEOS) and tissue resident eosinophils (rEOS) in Chronic rhinosinusitis with nasal polyps (CRSwNP). Simmalee et al. discussed different populations of eosinophils and how an imbalance of iEOS and rEOS exacerbates the type 2 endotype of CRSwNP, both due to eosinophil mechanisms and by signaling to other immune cells. The authors also placed a heavy focus on the potential impact of CRSwNP treatments which further exploration of eosinophil population may offer; this includes anti-eosinophilia drug treatment or combinations and using subtypes of eosinophils as biomarkers in clinical disease management and as predictor variables in models.

In their perspective article, Jin et al. explore the role of circulating immune complexes in the development of arthritis among patients with inflammatory bowel disease (IBD). They highlight how increased intestinal permeability, or “leaky gut”, in IBD allows microbial products to enter the circulation. This translocation facilitates the formation and systemic distribution of immune complexes, which may deposit in joints and trigger inflammatory responses. The authors further discuss potential strategies to restore gut barrier integrity, emphasizing the therapeutic potential of butyrate, a short-chain fatty acid known to support epithelial health and reduce permeability. This work underscores the gut-joint axis as a possible target for preventing extraintestinal complications of IBD.

The research article of Mobbs et al. presents a novel 3D human mucosal model that replicates key pathological features of IBD, including epithelial barrier dysfunction, immune activation, and stromal remodeling. The model integrates epithelial cells, stromal fibroblasts, and immune elements within a scaffold, simulating disease-relevant tissue architecture. Upon stimulation, it exhibits increased permeability, altered tight junction gene expression, and heightened inflammatory cytokine production. Notably, matrix metalloproteinases (MMPs)—especially MMP-9—are upregulated, mirroring patterns seen in Crohn’s disease tissues. TNF-α drives this MMP upregulation, underscoring its role in stromal remodeling. Histological analyses confirm that the model closely resembles changes observed in patient biopsies. This IBD model provides a physiologically relevant platform to accelerate the development of targeted therapies for diverse forms of IBD, it is a powerful tool which enhances translational efforts and therapeutic screening in a controlled, human-like setting.

Staying on the subject of the intestinal epithelial barrier, the original research by Yang et al. identified that deficiency of *Btbd8* in mice reduces intestinal barrier permeability associated with IBD. Yang et al. characterized a *Btbd8* knockout (KO) mouse model and demonstrated that the absence of *Btbd8* is associated with milder IBD phenotypes, when IBD is chemically induced. Further, proteomic and transcriptomics analyses confirmed that *Btbd8* deficiency is linked to increased expression of key tight junction proteins, such as ZO-1. The authors were able to show that mechanistically, *Btbd8* deficiency reduces the endocytosis of tight junction proteins, thereby preserving barrier integrity. Additionally, *Btbd8* deficiency promotes the proliferation of intestinal stem cells and goblet cells—the former supporting tissue regeneration and the latter enhancing mucosal defense by secreting mucus. Finally, the absence of *Btbd8* may also limit infiltration or activation of pro-inflammatory immune cells in the gut. Overall, Yang et al. highlight *Btbd8* as a promising therapeutic target for IBD.

The review article by Lu et al. examines the role which epithelial barrier dysfunctions play in allergic diseases such as asthma, atopic dermatitis, allergic rhinitis, and food allergy. The article introduces how genetic predispositions, allergens, pollutants, and microbial imbalances degrade tight and adherens junctions, increasing tissue permeability and triggering type 2 immune responses. Lu et al. outlines how allergens and pathogens directly damage epithelial structure and describes each of their unique physical or biochemical mechanisms of breaching the epithelial barrier tissue. Moreover, the authors discussed means to evaluate and quantify epithelial barrier function and introduced intervention methods such as immune modulation via allergen immunotherapy, topical barrier restoration, nutritional interventions, and microbiota regulation, which aims to restore the epithelial barrier’s function in the context of allergic diseases.

The original research article by Webb et al. investigated the *in vivo* effects of HIV pre-exposure prophylaxis (PrEP) on the expression of over 100 tight junction (TJ) genes in foreskin tissue. Using RNA sequencing of circumcised tissue from volunteers treated with two different PrEP regimens, the study generated a comprehensive TJ gene expression profile. Results showed minimal differences in TJ gene expression between PrEP-treated and control groups, suggesting that oral PrEP does not compromise the mucosal epithelial barrier. Additionally, while TJ genes correlated with cytokine signaling, PrEP had no significant impact on inflammatory gene expression. These findings support the safety of oral PrEP in preserving epithelial integrity and encourage further investigation into its broader biological effects.

In conclusion, this Research Topic highlights recent advancements in the study of barrier tissues across multiple organ systems. This topic presents clinically relevant discussions and insights into how dysfunctional barrier tissues may participate with health disorders. This topic aims to encourage further studies on barrier tissues and translational research, showcasing innovative models for studying barrier function, progress in biomarker discovery, and the development of novel diagnostic and treatment strategies.

